# The impact of white matter lesions on seizure recurrence after first epileptic seizures in the elderly: a prospective study

**DOI:** 10.1186/s42466-025-00391-2

**Published:** 2025-05-20

**Authors:** Jenny Weil, Louise Linka, Mariana Gurschi, Seyyid Abdulkerim Kidik, Alena Fuchs, Rebecca Schoenfeldt, Felix Zahnert, Leona Möller, Katja Menzler, André Kemmling, Susanne Knake, Lena Habermehl

**Affiliations:** 1 Department of Neurology, Epilepsy Center Hessen, Philipps-University Marburg, 35043 BaldingerstraßeMarburg, Germany; 2 Center for Neuroradiology, Philipps-University Marburg, Marburg, Germany; 3 Center for Mind, Brain and Behavior, CMBB, Philipps-University Marburg, Marburg, Germany; 4 Department of Neurology, University Hospitals of Cleveland Medical Centre, Cleveland, OH USA

**Keywords:** First seizure, Seizure recurrence, White matter lesions, Elderly, ARWMC rating scale

## Abstract

**Background:**

Despite considerable previous research, to what degree white matter lesions (WML) may be epileptogenic remains unclear. Therefore, the decision of initiating treatment with antiseizure medication (ASM) can be challenging in patients with only WML on neuroimaging. In this prospective study we assessed whether the prevalence, localization or severity of WML impact the risk of seizure recurrence in patients aged 60 years or older after first-time seizures.

**Methods:**

Data was analyzed from 168 patients, aged ≥ 60 years-old who had experienced a previous unprovoked seizure and had either a potentially epileptogenic lesion or WML on neuroimaging. The frequency of seizure recurrence was documented after 6, 12, and 24 months. Pearson´s chi-square test of independence (categorical variables) and the independent Student´s t-test (continuous variables) were used to analyze intergroup differences. Binary logistic regressions were calculated to examine the influence of WML locations as a predictor of seizure recurrence. Kaplan–Meier survival analyses and log-rank statistics were performed to determine the cumulative recurrence rates between the groups.

**Results:**

Fifteen patients had only potentially epileptogenic lesions on neuroimaging (EPI) and 93 showed WML only (OWML). Sixty patients showed both of them on neuroimaging (EWML). Frontal and parieto-occipital were the predominant WML locations. Neither severity nor location of WML had a significant impact on recurrence rates. The two-year cumulative probability of becoming seizure-free was significantly lower in the EPI group compared to the EWML (*χ*^*2*^ [1] = 4.425, *p* = 0.035) and the OWML group (*χ*^*2*^ [1] = 13.094, *p* < 0.001). A significant association between interictal epileptiform discharges in EEG and seizure recurrence was found in OWML patients (*p* = 0.004).

**Conclusion:**

We could not find any association between prevalence, severity or location of WML and seizure recurrence after first seizures in the elderly. Therefore, treatment with ASM should be started with caution in those patients. Our results show a trend of WML not having epileptogenic potential, but further studies are needed to get better evidence.

*Trial registration*: ClinicalTrials.gov Protocol Registration and Results, NCT06836687, AZ 199/17, release: 03/19/2024 retrospectively registered. https://register.clinicaltrials.gov/prs/beta/studies/S000EBC700000025/recordSummary

## Background

The incidence of unprovoked seizures and epilepsy in the elderly is higher than in any other age group [14, 24]. Late-onset epilepsy (LOE), defined as a new diagnosis of epilepsy at age 60 years or older, is mostly caused by stroke and other cerebrovascular diseases [4, 22]. These are often associated with chronic hypertension and other vascular risk factors, which are highly prevalent in the elderly [9].

However, in many cases, there is no evidence of a potentially epileptogenic lesion on brain imaging, making the cause of new-onset seizures unclear [14, 15].

In addition, it remains unknown if some non-specific neuroimaging findings have epileptogenic properties, e.g. global atrophy or cerebral small vessel disease (CSVD) [8, 22].

White matter lesions (WML), which are biomarkers of CSVD [30] and which are associated with an increased risk of subsequent stroke, dementia, and higher mortality [5] are a common neuroimaging finding in many older individuals.

However, WML are significantly more common in patients with LOE compared to healthy controls [22]. The relationship between WML and LOE has been controversial. Some studies suggest that WML occur more frequently during the course of a long-term epilepsy diagnosis [11, 12, 21]. Research by Johnson et al. suggests that the prevalence of WML is increased even before the first seizure and diagnosis of LOE, raising the question of whether WML cause LOE [17]. Furthermore, the increased prevalence may be due to a completely different context. Overall, the role of WML and CSVD in epileptogenicity and seizure recurrence is not fully understood [8].

Few studies have focused on patients with first-onset seizures and, to date, no prospective studies have focused on the prognosis or treatment of patients with WML-related seizures [6, 8]. Especially in patients with first-onset seizures, determining the epileptogenicity of WML is important in assessing the risk of seizure recurrence and guiding treatment with anti-seizure medication (ASM).

In this prospective study, we assessed the risk of seizure recurrence after first unprovoked seizures in patients aged 60 or older with WML, as compared to patients with potentially epileptogenic lesions. To answer this question all our patients underwent MR- or CT-Imaging during their first seizure hospitalization. We detected potentially epileptogenic lesions and categorized WML with the “age-related white matter changes (ARWMC) rating scale” [29]. The seizure recurrence rates were documented over 2 years after the first seizure.

## Methods

The sample was composed of patients aged 60 years or older presenting with a first epileptic seizure at the University Hospital Marburg, Germany from March 2018 to March 2023. Patients with provoked or acute symptomatic seizures were excluded. All demographic and clinical data were prospectively collected during the first seizure hospitalization. Clinical data included semiology, EEG, CT and/or MRI results, diagnosis and initiation of ASM. Whether an MRI or CT lesion was classified as potentially epileptogenic was the decision of the neurologist in consultation with the neuroradiologist.

To quantify WML on CT and MRI, Wahlund et al. developed the ARWMC rating scale, which scores WML in four severity levels (0: no lesions, 1: focal, 2: incipient confluence, 3: diffuse confluence) and five locations (frontal, parieto-occipital, temporal, basal ganglia and infratentorial) for each hemisphere. Finally, the scores of each region were summed [29]. In our study, the incidence and ARWMC scale of WML were graded by a neuro-radiologist for all patients. To determine the frequency of seizure recurrence, follow-up visits were scheduled at 6, 12 and 24 months. Patients who were unable to attend their follow-up visits were contacted by telephone. Patients were considered as “lost to follow-up” if we did not have data for that time period. Reasons for this were death, withdrawal from the study at the patient's request, failure to attend appointments and lack of telephone availability on three attempts.

### Statistical analysis

All statistical analyses were performed using IBM SPSS Statistics Version 29.

Results were considered significant at *α* = 0.05. Descriptive and frequency statistics were used to examine the differences in clinical characteristics in patients with WML and such with presumed epileptogenic lesions as well as in patients with different ARWMC scores. The results were given as absolute numbers with percentages, means (*m*) and standard deviation (*SD*). We used Pearson´s chi-square test of independence for analyzing intergroup differences of categorical variables and the independent Student´s t-test for continuous variables. Binary logistic regressions were calculated to investigate the influence of WML locations as a predictor of seizure recurrence. Kaplan–Meier survival analyses and log-rank statistics were performed to determine the cumulative recurrence rates of the different groups.

## Results

### Study population characteristics

Of 215 total patients, seven were excluded due to having non-epileptic seizure like events (e.g. syncope, psychogenic seizure) and 33 were excluded due to having a provoked or acute symptomatic seizure (e.g. acute stroke, metabolic/toxic, brain injury). Of the remaining 175 patients who had a first unprovoked seizure, 75 had a potentially epileptogenic lesion on neuroimaging (Table 1). Of these 60 patients had WML (ARWMC ≥ 1) on neuroimaging as well (EWML group) and 15 patients had presumed epileptogenic lesions only (EPI group).

**Table 1:** Sample characteristics in patients with unprovoked seizure (n = 168)

	EPI (*n* = 15)		EWML (n = 60)		OWML (*n* = 93)	
Characteristics	*n*	%	*N*	*%*	*n*	%
Sex						
Female	*3*	*20.0*	*27*	*45.0*	*50*	*53.8*
Male	*12*	*80.0*	*33*	*55.0*	*43*	*46.2*
Seizure recurrence reported*	*9*	*64.3*	*13*	*30.2*	*11*	*18.0*
Diagnosis of epilepsy	*15*	*100.0*	*57*	*95.0*	*63*	*67.7*
Antiseizure medication started	*15*	*100.0*	*58*	*96.7*	*70*	*75.3*
Status epilepticus as first seizure	*4*	*26.7*	*16*	*26.7*	*17*	*18.3*
Death during study-period	*3*	*20.0*	*20*	*33.3*	*21*	*22.6*
Neuroimaging						
MRI	*5*	*33.3*	*44*	*73.3*	*70*	*75.3*
CT**	*10*	*66.7*	*16*	*26.7*	*23*	*24.7*
Potentially epileptogenic findings***						
Postischemic lesion	*7*	*46.7*	*31*	*51.7*	*–*	*–*
Posthemorrhagic lesion	*5*	*33.3*	*14*	*23.3*	*–*	*–*
Tumor/Metastasis	*3*	*20.0*	*23*	*38.3*	*–*	*–*
Hippocampal sclerosis	*1*	*6.7*	*3*	*5.0*	*–*	*–*
Cortical dysplasia	*0*	*0.0*	*1*	*1.7*	*–*	*–*
Cavernoma	*1*	*6.7*	*0*	*0.0*	*–*	*–*
EEG						
Epileptiform discharge	*1*	*6.7*	*15*	*25.0*	*25*	*26.9*
No epileptiform findings	*14*	*93.3*	*44*	*73.3*	*67*	*72.0*
No EEG	*0*	*0.0*	*1*	*1.7*	*1*	*1.1*

EPI = only presumed epileptogenic lesions on neuroimaging, EWML = presumed epileptogenic lesions and white matter lesions on neuroimaging, OWML = only white matter lesions on neuroimaging

^*^EPI: *n* = 14, EWML: *n* = 43, OWML: *n* = 61, lost to follow-up: *n* = 50

^**^CT was only assessed by patients without MRI

^***^In the EPI group two of 15 patients got two potentially epileptogenic findings and in the EWML group 12 of 60 patients got two potentially epileptogenic findings

Among 100 patients without epileptogenic lesions, we found WML in 93 patients (OWML group) and other non-epileptogenic neuroimaging findings in seven patients of which six patients had normal neuroimaging findings and one patient had global atrophy. We excluded these seven patients with normal/unspecific neuroimaging findings from our further analysis. Other non-epileptogenic neuroimaging findings (e.g. atrophy) were not independently analyzed in our statistics.

Table 1 provides an overview of sample characteristics for each group. The mean age of our sample was 76.0 years (*SD* = 8.9, *range* = 60–95). Treatment with ASM was initiated in 143 patients (85.1) after the first seizure. No significant age difference (*p* = 0.314) was found between patients in which ASM was started (*m* = 75.7 years, *SD* = 8.7) and those in which no treatment was started (*m* = 77.7 years, *SD* = 9.8).

All patients received at least one type of neuroimaging during their hospitalization. 70.8% (119 patients) received a brain MRI. CT scans were used for analysis if MRI was unavailable (29.2%, 49 patients).

**Fig. 1:**
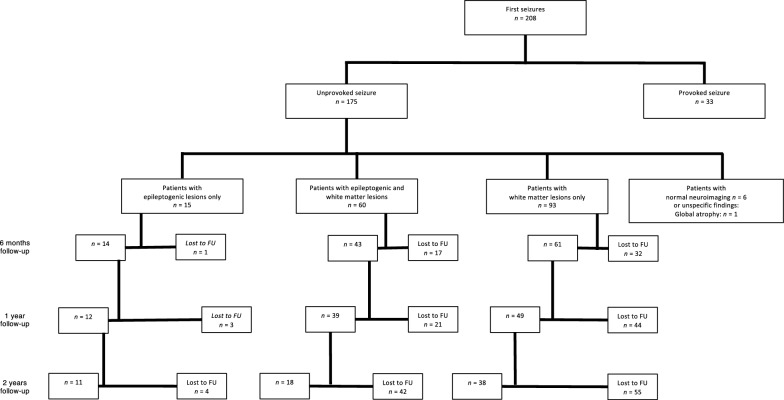
Sample of patients at each follow-up (FU) examination

**Fig. 2:**
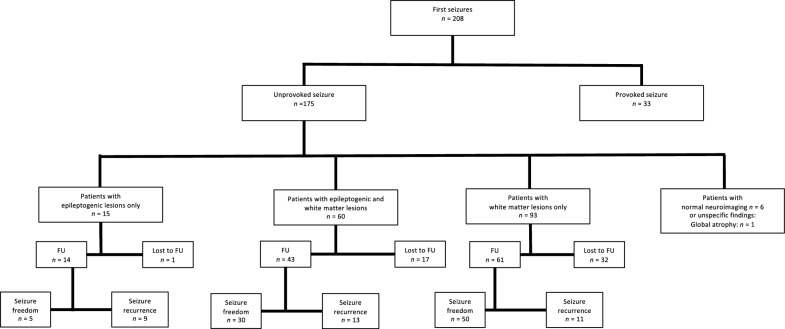
Neuroimaging findings and seizure-recurrence rates. FU = all patients with at least one follow-up

Figure 1 shows the follow-up rates for each follow-up examination (FU) at six months, one year and two years. Figure 2 illustrates the total follow-up rates and rates of seizure recurrence for the three groups. Data for 118 patients (70.2%) was obtained at least at one follow-up appointment. 50 patients (29.8%) were completely lost to follow-up, of which 28 (56.0%) died before the first follow-up.

### Patients with white matter lesions

Overall, we analyzed two groups of patients with WML: The EWML group with presumed epileptogenic lesions and WML (n = 60) and the OWML with WML only (n = 93).

**Fig. 3:**
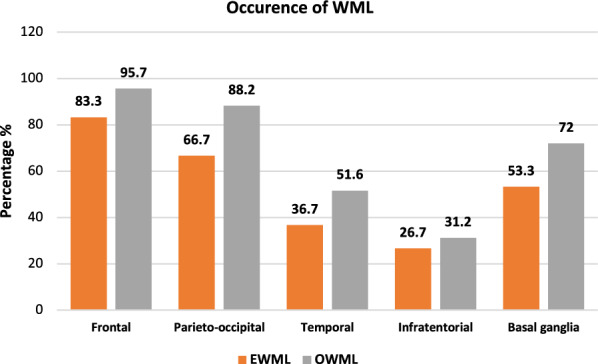
Occurrence rates of white matter lesions (WML) for each location (in %) in all patients with WML (ARWMC score ≥ 1),* n* = 153

Figure 3 shows the prevalence rates of WML for each location in all patients with WML.

(*n* = 153, ARWMC ≥ 1). Overall, frontal (OWML: 95.7% and EWML: 83.3%) and parieto-occipital (OWML: 88.2% and EWML: 66.7%) showed the highest prevalence. Anyway, 51.6% of the OWML patients had WML in the temporal lobes.

Of 153 patients with WML, we had follow-up data for 104 patients (67.9%). Eleven of the OWML patients (18.0%) and 13 of the EWML (30.2%) reported seizure recurrence.

Regarding whether the severity of WML, affected the outcome, Table 2 shows the means and *SD* of the ARWMC scale for each location and in total for patients with seizure recurrence (*n* = 24) and seizure freedom (*n* = 80). The frontal and parieto-occipital regions had the highest mean scores overall. The median number of regions with WML was six in the OWML group and four in the EWML group, while only 2 patients (3.3%) of the EWML group and 19 patients (20.4%) of the OWML group had WML in all ten regions.

**Table 2:** Means *(m)* and Standard Deviation *(SD)* of ARWMC scale for each location

	OWML Group				EWML Group			
	Seizure recurrence (*n* = 11)		Seizure freedom (*n* = 50)		Seizure recurrence (*n* = 13)		Seizure freedom (*n* = 30)	
Location	*m*	*SD*	*m*	*SD*	*m*	*SD*	*m*	*SD*
Frontal								
Left	1.91	0.83	1.4	0.9	1.38	0.87	1.07	0.98
Right	1.82	0.75	1.48	0.89	1.00	0.91	1.1	1.03
Parieto-occipital								
Left	1.64	0.81	1.3	0.99	1.00	1.08	0.73	0.78
Right	1.64	0.81	1.24	1.00	0.92	1.12	0.5	0.68
Temporal								
Left	0.73	0.79	0.42	0.61	0.62	0.77	0.27	0.45
Right	0.73	0.79	0.42	0.61	0.31	0.48	0.27	0.45
Basal ganglia								
Left	1.00	0.63	0.8	0.78	0.77	0.83	0.7	0.88
Right	1.00	0.63	0.66	0.75	0.69	0.85	0.7	0.84
Infratentorial								
Left	0.54	0.69	0.26	0.63	0.46	0.88	0.2	0.48
Right	0.45	0.69	0.22	0.55	0.38	0.87	0.27	0.52
ARWMC in total	11.45	5.84	8.2	5.89	7.54	6.41	5.8	4.73

*OWML* Only white matter lesions (*n* = 61)

*EWML* Epileptogenic and white matter lesions (*n* = 43)

### OWML group

Of 93 patients with OWML we had follow up data for 61 patients (65.6%). The eleven seizure-recurrent patients showed a mean total ARWMC score of 11.45 (*SD* = 5.84, *range* = 3–22), whereas the seizure-free patients (n = 50) had a mean total score of 8.2 (*SD* = 5.89, *range* = 1–26). Overall, the comparison of the two groups using an independent Student´s t-test showed no significance (*t*(59) = − 1,662, *p* = 0.102).

Treatment with ASM was initiated in 49 of 61 patients (80.3%), including all eleven patients with seizure recurrence. A total of 38 patients (77.6%) became seizure-free with the first ASM. In all patients with recurrent seizures, recurrence was reported within the first year after the initial seizure. Although no epileptogenic lesions were detected on neuroimaging in the OWML group, we found epileptiform discharges on EEG in 26.9% of them.

Furthermore, we found that seven (63.6%) of the patients with seizure recurrence had interictal epileptiform discharges (IED) on EEG. Of the four patients (36.4%) without IED, three showed generalized or regional slowing and only one patient had a normal EEG. In comparison, seizure free patients showed IED in 10 out of 49 cases (20.4%), while one patient did not receive EEG. A chi-square test of independence revealed a significant association between EEG findings and seizure recurrence in patients with WML only (*χ*^*2*^ [1, n = 60] = 8.267, *p* = 0.004, *φ* = − 0.371) indicating a medium effect size.

In two binary logistic regression models (one for each hemisphere: left: *χ*^*2*^ [5, *n* = 61] = 3.768, *p* = 0.583; right: *χ*^*2*^ [5, *n* = 61] = 2.861, *p* = 0.721) with seizure recurrence as dependent variable and the different ARWMC locations (frontal, parieto-occipital, temporal, infratentorial and basal ganglia) as independent variables, we found no location that was independently associated with seizure recurrence (all *p* > 0.05).

### EWML group

In the EWML group we had follow-up data for 43 of 60 patients (71.7%) of which 13 had at least one recurrent seizure (30.2%). The seizure-free patients showed a mean total ARWMC score of 5.8 (*SD* = 4.73, *range* = 1–16), whereas the 13 seizure-recurrent patients had a mean total score of 7.54 (*SD* = 6.41, *range* = 1–23). An independent Student´s t-test for the comparison of both groups showed no significance (*t*(41) = − 0,992, *p* = 0.327).

Treatment with ASM was initiated in 41 of 43 patients (95.3%), including all 13 patients with seizure recurrence. A total of 28 patients (68.3%) became seizure-free with the first ASM. Of the seizure recurrent patients only one (7.7%) had interictal epileptiform discharges (IED) on EEG. We again performed two binary logistic regression models (one for each hemisphere: left: *χ*^*2*^ [5, *n* = 43] = 4.157, *p* = 0.527; right: *χ*^*2*^ [5, *n* = 43] = 3.002, *p* = 0.7) with seizure recurrence as dependent variable and the different ARWMC locations (frontal, parieto-occipital, temporal, infratentorial and basal ganglia) as independent variables and found no location that was independently associated with seizure recurrence (all *p* > 0.05) in the EWML group either.

### Comparison of EPI, OWML and EWML group

**Fig. 4:**
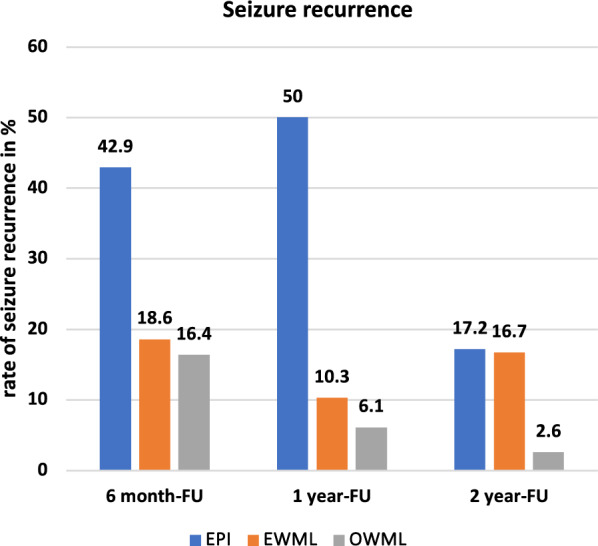
Recurrence rates (in %) at each follow-up (FU) examination for patients with presumed epileptogenic lesions only (EPI), patients with presumed epileptogenic and white matter lesions (EWML) and patients white matter lesions only (OWML) on neuroimaging

Comparing the OWML group and the EWML group with the EPI group, Fig. 4 shows the exact percentages of recurrence rates for each follow-up examination.

In the EPI group we had follow-up data for 14 out of 15 patients (93.3%). Of these 9 (64.3%) reported seizure recurrence. A chi-square test of independence with Bonferroni correction revealed a significant difference in recurrence rates for the three groups 1 year after the initial seizure (*χ*^*2*^ [2, *n* = 100] = 16.834, *p* < 0.001, *φ* = 0.41). After the first year the EPI group showed a recurrence rate of 50.0%, while seizure recurrence in the EWML group was only detected in 10.3%. The OWML group showed seizure recurrence only in 6.1% of the patients.

**Fig. 5:**
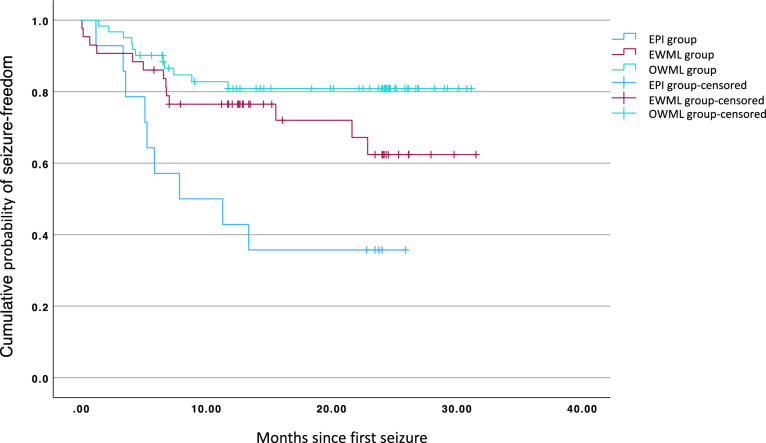
Kaplan–Meier curve on recurrence rate after first seizure for patients with epileptogenic findings only (EPI group), patients with epileptogenic and white matter lesions (EWML group) and patients with white matter lesions only (OWML group) in months

Additionally, Fig. 5 illustrates that the two-year cumulative probability of seizure recurrence differs significantly between the three groups (log-rank: *χ*^*2*^ [2] = 12.546, *p* = 0.002). Patients were censored at the time they were lost to follow-up. Pairwise post-hoc log-rank tests revealed a statistically significant difference for the probability of becoming seizure-free between the OWML and the EPI group (*χ*^*2*^ [1] = 13.094, *p* < 0.001), as well as between the EWML and the EPI group (*χ*^*2*^ [1] = 4.425, *p* = 0.035), indicating not only that the OWML group had a significantly lower risk for seizure recurrence compared to the EPI group, but also that the patient group with presumed epileptogenic lesions and WML had a lower risk for seizure recurrence compared to the group with presumed epileptogenic lesions only. There was no significant difference in seizure recurrence rates between the OWML and the EWML group (*χ*^*2*^ [1] = 2.087, *p* = *0.149*).

## Discussion

In the present study, we prospectively analyzed data from 168 patients aged 60 years or older to investigate whether WML have an impact on seizure recurrence after a first unprovoked seizure in the elderly. Fifteen of our patients showed only potentially epileptogenic lesions, 93 patients had only WML and 60 patients showed both on brain imaging. Neither the severity of WML, as assessed by the ARWMC scale, nor the location of WML had a significant effect on recurrence rates. Similarly, Green et al. studied first-seizure patients aged 60 years and older and concluded that in those with CSVD, the severity did not predict seizure recurrence [10]. Another study confirmed these findings in patients 60 years or older with new-onset epilepsy of structural or unknown etiology. In contrast, the pure presence of leukoaraiosis alone was associated with a lower likelihood of becoming seizure-free [27]. Uslu et al. found that WML were not associated with seizure frequency in adult patients with a diagnosis of epilepsy [28].

However, in our sample, the group with presumed epileptogenic lesions had a significantly lower likelihood to achieve seizure freedom compared to all patients with WML. The two-year cumulative risk of seizure recurrence was significantly higher in the EPI group than in the EWML group (*p* = 0.035) and OWML group (*p* < 0.001). This suggests, as expected, that WML might not have as high epileptogenicity as epileptogenic lesions such as cortical strokes or brain tumors.

Since most of our patients were treated with ASM, we cannot exclude the possibility that the differences in recurrence rates between the groups would have been lower if ASM had not been started. This means that the data from our study cannot completely rule out that WML could have an epileptogenic potential. But it seems certain that the risk of drug-refractory epilepsy is lower in the WML groups, which also suggests that WML are less likely to be epileptogenic than conventional epileptogenic lesions. Previous studies are consistent with the suggestion that WML alone do not explain the etiology of LOE and that the origin of seizures without structural lesions may be a combination of several factors [1]. Interestingly, however, the patients with assumed epileptogenic lesions and WML (EWML) also have a significantly lower risk of recurrence than the group with assumed epileptogenic lesions only. To our knowledge, no protective effect of WML has been demonstrated to date. It seems more likely that the epileptogenic lesions of patients with WML are mainly strokes, which show a relatively low epileptogenicity [7, 16].

Regarding the distribution of WML in our study population, they are highly prevalent in the frontal and parieto-occipital lobes. This finding is consistent with further literature. Zhang et al. described that ischemic WML occur mainly in the frontal and parieto-occipital lobe and age is independently associated with frontal WML [31]. In addition, seizures were one of the most common symptoms of frontal and parieto-occipital WML and older age was associated with a higher WML burden in a cohort of patients with cerebral microangiopathy aged 45–95 years. In these 90.7% of the cerebral microangiopathy was localized in frontal and parieto-occipital regions [23]. Furthermore, WML in the basal ganglia and infratentorial area appear to be a marker of hypertension [31], which is also a common comorbidity in the elderly. This suggests that the high rate of frontal, parieto-occipital and basal ganglia WML in our patients may be primarily due to age and age-related comorbidities.

A significant association was found between EEG findings and seizure recurrence in OWML patients, with 90.9% of seizure-recurrent patients having a pathologic EEG with IED (63.6%) or regional slowing (27.3%). Since the presence of epileptiform activity on EEG is known to be one of the major biomarker for seizure recurrence [3], this suggests that recurrence in our OWML patients may be predicted by pathologic EEG findings rather than the presence of WML only. We should consider this before diagnosing epilepsy in patients with WML on MRI but normal EEG and before starting with ASM.

Most of the literature to date is based on patients with a diagnosis of epilepsy and not just on first-onset seizure patients. Arabi et al. showed a WML prevalence of 69% in patients ≥ 60 years and a mean age of 72 years (*range*: 60.5–86.5) with new onset cryptogenic epilepsy [2]. Mao et al. had a WML prevalence of 66.4% in patients with chronic epilepsy in a study population much younger than ours [21], but described that age was positively associated with a higher prevalence of WML (higher ARWMC score) which is confirmed by several studies [25, 26, 28]. The overall prevalence of WML in our cohort (mean age = 75.8 years) is very high at 87.4% compared to the other studies mentioned. There is also evidence that a higher degree of WML earlier in life is associated with a higher risk of seizures and late-onset epilepsy in the elderly [11, 17]. This hypothesis is supported by the high prevalence in our elderly first-onset seizure patients.

In addition, some authors have hypothesized that WML may also represent secondary effects of seizures and ASM [13]. Our results, which show that WML are highly prevalent in first-seizure patients prior to treatment initiation, do not support this hypothesis.

In general, older patients are more likely to be treated after a first seizure, although age alone is not a predictor for seizure recurrence [18]. Linka et al. have shown that ASM treatment has a significant benefit in reducing recurrence in elderly patients with first-onset seizures [20]. Nevertheless, the treatment of epilepsy in elderly patients remains challenging due to factors such as changes in pharmacokinetics, polytherapy and susceptibility to adverse drug reactions [19]. In light of our results, initiation of treatment with ASM in elderly patients with first-onset seizures and “only” WML on imaging should be carefully considered.

The major strength of this study is its prospective design. However, there are some limitations including the small sample size and the treatment with an ASM as described above. In addition, not all patients underwent MRI. Even though the ARWMC scale is designed for both CT and MRI, MRI performs better in detecting small lesions [29]. The use of a 3 Tesla MRI as the standard scanner would likely have detected even more lesions on MRI than the 1.5 Tesla MRIs used. Due to the study’s observational nature, there is no comparison group with an unremarkable MRI. Furthermore, the overall lost-to-follow-up rate in our study is relatively high (29.8%), which may be due to several reasons. One was the COVID-19 pandemic, which prevented many patients from coming to the hospital for their follow-up visits. Because of the age of the study population, it was difficult for many patients to attend follow-up visits. This resulted in a higher percentage of study discontinuations compared to younger individuals, especially when seizures had ceased. This means that the rate of seizure freedom may be underestimated. Another reason is that the mortality in our study population is relatively high (26.2%) due to the advanced age.

In addition, due to the ongoing design of the study, some patients were not able to attend all follow-up visits. Therefore, it is important to re-evaluate our results in further studies with larger untreated study populations of patients with first-onset seizures.

## Conclusion

We could not find any association between the severity or localization of WML and seizure recurrence after first unprovoked seizures in elderly patients. However, the two-year cumulative probability of seizure recurrence was significantly higher in patients with epileptogenic lesions only than in those with WML. The results should be considered by clinicians and are more of an argument against initiating treatment with an ASM in older patients with first epileptic seizure without lesions other than WML and without pathologic EEG findings.

## Data Availability

The data sets used and/or analyzed during the current study are available from the corresponding author upon reasonable request.

## Abbreviations

ARWMC: Age-related white matter changes
ASM: Antiseizure medication
CSVD: Cerebral small vessel disease
EPI: Presumed epileptogenic lesions
EWML: Presumed epileptogenic lesions and white matter lesions
FU: Follow-up
IED: Interictal epileptiform discharges
LOE: Late-onset epilepsy
OWML: Only White matter lesions
WML: White matter lesions
