# Correction: The impact of white matter lesions on seizure recurrence after first epileptic seizures in the elderly: a prospective study

**DOI:** 10.1186/s42466-025-00397-w

**Published:** 2025-06-11

**Authors:** Jenny Weil, Louise Linka, Mariana Gurschi, Seyyid Abdulkerim Kidik, Alena Fuchs, Rebecca Schoenfeldt, Felix Zahnert, Leona Möller, Katja Menzler, André Kemmling, Susanne Knake, Lena Habermehl

**Affiliations:** 1 Department of Neurology, Epilepsy Center Hessen, Philipps-University Marburg, 35043 BaldingerstraßeMarburg, Germany; 2 Center for Neuroradiology, Philipps-University Marburg, Marburg, Germany; 3 Center for Mind, Brain and Behavior, CMBB, Philipps-University Marburg, Marburg, Germany; 4 Department of Neurology, University Hospitals of Cleveland Medical Centre, Cleveland, OH USA

**Correction: Neurological Research and Practice (2025) 7:36**

10.1186/s42466-025-00391-2

Due to a typesetting mistake, the values in the rows ‘Tumor/Metastasis’ and ‘Hippocampal sclerosis’ in Table 1 were incorrectly given.

**Table 1:** Sample characteristics in patients with unprovoked seizure (n = 168)

	EPI (*n* = 15)		EWML (n = 60)		OWML (*n* = 93)	
Characteristics	*n*	%	*N*	*%*	*n*	%
Sex						
Female	*3*	*20.0*	*27*	*45.0*	*50*	*53.8*
Male	*12*	*80.0*	*33*	*55.0*	*43*	*46.2*
Seizure recurrence reported*	*9*	*64.3*	*13*	*30.2*	*11*	*18.0*
Diagnosis of epilepsy	*15*	*100.0*	*57*	*95.0*	*63*	*67.7*
Antiseizure medication started	*15*	*100.0*	*58*	*96.7*	*70*	*75.3*
Status epilepticus as first seizure	*4*	*26.7*	*16*	*26.7*	*17*	*18.3*
Death during study-period	*3*	*20.0*	*20*	*33.3*	*21*	*22.6*
Neuroimaging						
MRI	*5*	*33.3*	*44*	*73.3*	*70*	*75.3*
CT**	*10*	*66.7*	*16*	*26.7*	*23*	*24.7*
Potentially epileptogenic findings***						
Postischemic lesion	*7*	*46.7*	*31*	*51.7*	*–*	*–*
Posthemorrhagic lesion	*5*	*33.3*	*14*	*23.3*	*–*	*–*
Tumor/Metastasis	*3*	*20.0*	*20*	*33.3*	*21*	*22.6*
Hippocampal sclerosis	*1*	*6.7*	*31*	*51.7*	*–*	*–*
Cortical dysplasia	*0*	*0.0*	*1*	*1.7*	*–*	*–*
Cavernoma	*1*	*6.7*	*0*	*0.0*	*–*	*–*
EEG						
Epileptiform discharge	*1*	*6.7*	*15*	*25.0*	*25*	*26.9*
No epileptiform findings	*14*	*93.3*	*44*	*73.3*	*67*	*72.0*
No EEG	*0*	*0.0*	*1*	*1.7*	*1*	*1.1*

EPI = only presumed epileptogenic lesions on neuroimaging, *EWML* presumed epileptogenic lesions and white matter lesions on neuroimaging, OWML = only white matter lesions on neuroimaging

^*^EPI: *n* = 14, EWML: *n* = 43, OWML: *n* = 61, lost to follow-up: *n* = 50

^**^CT was only assessed by patients without MRI

^***^In the EPI group two of 15 patients got two potentially epileptogenic findings and in the EWML group 12 of 60 patients got two potentially epileptogenic findings

Corrected Table 1:

**Table 1:** Sample characteristics in patients with unprovoked seizure (n = 168)

	EPI (*n* = 15)		EWML (n = 60)		OWML (*n* = 93)	
Characteristics	*n*	%	*N*	*%*	*n*	%
Sex						
Female	*3*	*20.0*	*27*	*45.0*	*50*	*53.8*
Male	*12*	*80.0*	*33*	*55.0*	*43*	*46.2*
Seizure recurrence reported*	*9*	*64.3*	*13*	*30.2*	*11*	*18.0*
Diagnosis of epilepsy	*15*	*100.0*	*57*	*95.0*	*63*	*67.7*
Antiseizure medication started	*15*	*100.0*	*58*	*96.7*	*70*	*75.3*
Status epilepticus as first seizure	*4*	*26.7*	*16*	*26.7*	*17*	*18.3*
Death during study-period	*3*	*20.0*	*20*	*33.3*	*21*	*22.6*
Neuroimaging						
MRI	*5*	*33.3*	*44*	*73.3*	*70*	*75.3*
CT**	*10*	*66.7*	*16*	*26.7*	*23*	*24.7*
Potentially epileptogenic findings***						
Postischemic lesion	*7*	*46.7*	*31*	*51.7*	*–*	*–*
Posthemorrhagic lesion	*5*	*33.3*	*14*	*23.3*	*–*	*–*
Tumor/Metastasis	*3*	*20.0*	*23*	*38.3*	-	-
Hippocampal sclerosis	*1*	*6.7*	*3*	*5.0*	*–*	*–*
Cortical dysplasia	*0*	*0.0*	*1*	*1.7*	*–*	*–*
Cavernoma	*1*	*6.7*	*0*	*0.0*	*–*	*–*
EEG						
Epileptiform discharge	*1*	*6.7*	*15*	*25.0*	*25*	*26.9*
No epileptiform findings	*14*	*93.3*	*44*	*73.3*	*67*	*72.0*
No EEG	*0*	*0.0*	*1*	*1.7*	*1*	*1.1*

EPI = only presumed epileptogenic lesions on neuroimaging, EWML = presumed epileptogenic lesions and white matter lesions on neuroimaging, OWML = only white matter lesions on neuroimaging

^*^EPI: *n* = 14, EWML: *n* = 43, OWML: *n* = 61, lost to follow-up: *n* = 50

^**^CT was only assessed by patients without MRI

^***^In the EPI group two of 15 patients got two potentially epileptogenic findings and in the EWML group 12 of 60 patients got two potentially epileptogenic findings

The original article has been corrected.

